# Assessment of Obesity and Associated Risk Factors of Diabesity in an Urban Population in Central India

**DOI:** 10.7759/cureus.39776

**Published:** 2023-05-31

**Authors:** Harshal G Mendhe, Sonali K Borkar, Mohammed Kamran Shaikh, Sonali G Choudhari

**Affiliations:** 1 Community Medicine, Datta Meghe Medical College, Datta Meghe Institute of Medical Sciences, Nagpur, IND; 2 School of Epidemiology & Public Health, Jawaharlal Nehru Medical College, Datta Meghe Institute of Medical Sciences, Wardha, IND

**Keywords:** waist circumference, waist hip ratio, body fat percentage, body mass index, diabesity

## Abstract

Background

Over the past 20 years, the prevalence of adult obesity has doubled. International awareness of the body mass index (BMI) as a benchmark for identifying and categorizing overweight and obesity has grown. This study was conducted to assess the socio-demographic factors of the study participants, assess the prevalence of obesity amongst the study subjects, find an association between risk factors and diabesity, and assess obesity using the percentage body fat and waist-hip ratio of study participants.

Methods

This study was undertaken among diabetes patients residing in the field practice area of the Urban Health and Training Centre (UHTC), Wadi, affiliated with the Datta Meghe Medical College, Nagpur, from July 2022 to September 2022. Two hundred and seventy-eight diabetic people were included as study participants. Systematic random sampling was used to identify study subjects visiting UHTC, Wadi. The World Health Organization's step-by-step approach to the surveillance of risk factors for chronic diseases served as the model for the questionnaire.

Results

Among the 278 diabetic study participants, the prevalence of generalized obesity was 76.61%. Obesity was more prevalent in subjects with a family history of diabetes. All hypertensive subjects were obese. Obesity was more prevalent among tobacco chewers. In obesity assessment using body fat percentage when compared with standard BMI, the sensitivity was found to be 84% and specificity was 48%.

Conclusion

Body fat percentage is a simple estimation that can identify obesity among diabetic individuals who are non-obese by BMI. We can change the behavior amongst non-obese diabetic individuals by giving health education, thereby reducing insulin resistance and improving compliance and adherence to the treatment.

## Introduction

Over the past 20 years, the prevalence of adult obesity has doubled. International awareness of body mass index (BMI) as a benchmark for identifying and categorizing overweight and obesity has grown. Environmental factors, such as diet, activity, and environmental toxins, may act as initiating factors or progression factors for type II diabetes mellitus (T2DM) [1]. In comparison to people of a healthy weight, obese and overweight people have seven times and three-fold higher risk of developing diabetes, respectively [2]. Additionally, it is predicted that excess weight is a contributing factor in about 90% of T2DM [3].

Insulin deficiency and resistance are principally responsible for the pathophysiology linking obesity and diabetes [4]. Up until March 2020, when the World Health Organization (WHO) declared coronavirus disease 2019 (COVID-19) to be a pandemic, diabesity, defined as the coexistence of T2DM and obesity, was thought to be the epidemic of the 21st century [5]. According to research by Cheung et al., obese Chinese T2DM patients consumed much more meat than non-obese T2DM patients [6]. In a study of T2DM patients in Belagavi, Karnataka, 58.68% were found to be obese [7].

The current study was undertaken among diabetes patients residing in the field practice area of the Urban Health and Training Centre (UHTC), Wadi, affiliated with Datta Meghe Medical College, Nagpur, India, to assess the socio-demographic factors among the study participants and the prevalence of obesity amongst them, to find an association between risk factors of chronic diseases with diabesity, and to assess obesity using percentage body fat and waist-hip ratio. 

## Materials and methods

Study Settings and study period

The field practice area of UHTC, Wadi, served as the site of the current cross-sectional study. The study was conducted from July 2022 to September 2022. The UHTC is located 8 km from the Datta Meghe Medical College, Nagpur, India and caters its services to a population of 58,000. The study was approved by the Institutional Ethics Committee of Shalinitai Meghe Hospital and Research Centre (approval number: DMMC(DU)/IEC/2022/78). The privacy of the respondents was respected and confidentiality was maintained up to the full extent.

Sample size calculation and sampling technique

The required sample size was 278, taking the 59% prevalence of obesity among T2DM patients in the study in Belagavi, Karnataka [7], with a 10% relative error and 95% confidence interval. Systematic random sampling was used to identify participants from among the patients who attended UHTC, Wadi. The Non-Communicable Disease Clinic register enrolled 537 diabetic patients from July 2019 to June 2022. The first participant was identified using a simple random technique, which turned out to be the fifth patient. Ater this, every alternate patient was included as a study participant till the desired sample size was attained.

Inclusion criteria are residents in the field practice area of UHTC, Wadi, having T2DM, and above 18 yrs of age. Exclusion criteria included: (i) pregnant and lactating women, (ii) type 1 DM patients, and (iii) patients refusing to participate or who did not give consent.

Data collection

Following the study participants' consent, data were gathered using a pretested structured interviewer-administered questionnaire. The WHO step-by-step approach for surveillance of risk factors for chronic diseases served as the model for the questionnaire [8]. We conducted in-person interviews with the diabetes respondents at their residences to gather data. To locate the study participants, a social worker's help was sought and they were visited at their homes.

The data contained behavioral, anthropometric, and clinical variables. Using standardized procedures and calibrated equipment, the study participants' height and weight were determined. Body weight was determined using a digital scale in a standing position, wearing just light clothing and no shoes, to the nearest 0.1 kg. It was ensured that the buttocks, scapula, and head of the participants were in contact with the stadiometer to accurately measure height in centimeters (cm) in an upright position. The average of two measurements was recorded. Weight in kilograms divided by the square of height in meters yielded the BMI. Obesity was assessed using the standard BMI classification given by WHO for the Indian population [9]. The socioeconomic status of the families was assessed using the Modified Kuppuswami Scale 2022 taking the Consumer Price Index for Industrial Workers, which was 129 in July 2022, the base year being 2016 [10].

Data analysis

Microsoft Excel (Microsoft Corporation, Redmond, Washington, United States) was used for the compilation of accurately collected data. This data was checked for completeness and correctness and analyzed using SPSS for Windows, Version 16.0 (Released 2007; SPSS Inc., Chicago, United States). Statistical tools applied were mean, percentages, confidence interval, and receiver operating characteristic (ROC) curve. The Chi-square test was used as a test of statistical significance.

Operational definitions

BMI

Using the WHO's conventional BMI classification for Indian populations, BMI of more than 25 kg/m^2^ is considered obese. 

Body Fat Percentage

The formulae for males and females are as follows [11]: For men = ([0.567 × WC in cm] + [0.101 × age in years]) - 31.8. For women = ([0. 438 × WC in cm] + [0. 221 × age in years]) - 9.4. As per the United States National Institutes of Health criteria for body fat percentage, less than 25% of body fat for men or 30% for women was considered obese [12].

Waist Hip Ratio

Waist-hip ratio of more than 1 in men and more than 0.85 in women was considered as obese.

Waist Circumference

A waist circumference of more than 90 cm in men and more than 80 cm in women was considered as obese.

## Results

Table 1 shows the socio-demographic characteristics of study participants. Amongst the 278 diabetic study participants, the prevalence of generalized obesity was 76.6%. In our study, 37.8% of study participants were in the age group of 51-60 years, while 32.7% were in the age group of 41-50 years. Females were 41.4% and 58.6% of participants were male. Centralized obesity was more among females (85%) as compared to males (63%).

**Table 1:** Socio-demographic characteristics of study participants

		Obese n (%)	Non-obese n (%)	Total N (%)
Age In Years	20-30	13 (4.7)	4 (1.4)	17 (6.1)
	31-40	32 (11.5)	9 (3.3)	41 (14.8)
	41-50	68 (24.5)	23 (8.3)	91 (32.7)
	51-60	81 (29.1)	24 (8.6)	105 (37.8)
	>60	19 (6.8)	5 (1.8)	24 (8.6)
Gender	Male	129 (46.4)	34 (12.2)	163 (58.6)
	Female	84 (30.2)	31 (11.2)	115 (41.4)
Religion	Hindu	129 (46.4)	43 (15.5)	172 (61.9)
	Muslim	84 (30.2)	22 (7.9)	106 (38.1)
Type of Family	Nuclear	128 (46)	32 (11.6)	160 (57.6)
	Joint	75 (27)	28 (10)	103 (37)
	Three generation	10 (3.6)	5 (1.8)	15 (5.4)
Education	Illiterate	62 (22.3)	17 (6.1)	79 (28.4)
	Primary School	17 (6.1)	4 (1.4)	21 (7.5)
	Middle School	34 (12.2)	13 (4.7)	47 (16.9)
	High School	53 (19.1)	8 (2.9)	61 (22)
	Intermediate	24 (8.6)	18 (6.5)	42 (15.1)
	Graduate/Postgraduate	23 (8.3)	5 (1.8)	28 (10.1)
Socio-economic status	Upper	24 (8.6)	17 (6.1)	41 (14.7)
	Upper middle	53 (19.1)	18 (6.5)	71 (25.6)
	Lower middle	36 (13)	2 (0.7)	38 (13.7)
	Upper lower	94 (33.8)	15 (5.4)	109 (39.2)
	Lower	6 (2.1)	13 (4.7)	19 (6.8)
Total		213 (76.6)	65 (23.4)	278 (100)

Among the study participants, 61.9% were Hindu and 38.1% were Muslim; 57.6% were from nuclear families and 37% were from joint families. A maximum of 28.4% of participants were illiterate, 22% completed high school education and only 10.1% were graduate or postgraduate. In our study, 39.2% belonged to upper lower socioeconomic status as per the Modified Kuppuswami scale, followed by 25.6% belonging to upper middle and 6.8% belonging to lower socioeconomic class.

Table 2 shows the association of different determinants with diabesity. Obesity was more prevalent amongst those diabetic subjects who had a family history of diabetes (96%) than those subjects who did not have a family history of diabetes (58%) and this was found to be statistically significant. All hypertensive participants were obese. Obesity was associated with hypertension and the association was found to be statistically significant. Obesity was more prevalent amongst those diabetic subjects who had a history of addiction (85%) than those who did not (15%) and this was found to be statistically significant. Obesity was more associated with alcoholic diabetic participants (93%) than non-alcoholic diabetic subjects (7%) and this was found to be statistically significant. Out of 39 diabetic bidi/cigarette smokers, 64% were obese and out of 239 diabetic non-smokers, 78.7% were obese. Thus, a direct association was not observed. Obesity was more prevalent in diabetic tobacco chewers excluding smokers (100%) and this was found to be statistically significant. Obesity was reversely associated with those participants who were doing daily physical exercise (27%) than those who were not exercising daily (73%) and this was found to be statistically significant. Obesity was not associated with the socioeconomic status of diabetic study participants.

**Table 2:** Association of different determinants with diabesity

Determinants		Obese n (%)	Non-obese n (%)	Total ( N=278)	Chi-square value	P value
Family history of DM	Yes	130 (96.3)	5 (3.7)	135	56.72	0.000
	No	83 (58)	60 (42)	143		
Having Hypertension	Yes	71 (100)	0 (0)	71	29.09	0.000
	No	142 (68.6)	65 (31.4)	207		
History of addiction	Yes	131 (84.5)	24 (15.5)	155	12.19	0.000
	No	82 (66.7)	41 (33.3)	123		
Alcoholic	Yes	70 (93.3)	5 (6.7)	75	16.01	0.000
	No	143 (70.4)	60 (29.6)	203		
Bidi/Cigarette smoker	Yes	25 (64.1)	14 (35.9)	39	3.967	0.046
	No	188 (78.7)	51 (21.3)	239		
Tobacco chewer	Yes	32 (100)	0 (0)	32	11.036	0.001
	No	181 (73.6)	65 (26.4)	246		
History of daily physical exercise	Yes	8 (26.7)	22 (73.3)	30	46.84	0.000
	No	205 (82.7)	43 (17.3)	248		
Socio-economic status	Higher	113 (75.3)	37 (24.7)	150	0.3	0.583
	Lower	100 (78.1)	28 (21.9)	128		

Table 3 depicts the sensitivity and specificity of body fat percentage in the estimation of obesity. In obesity assessment using body fat percentage when compared with standard BMI found the sensitivity 84% and specificity 48%.

**Table 3:** Sensitivity and specificity of body fat percentage in the estimation of obesity

		Obese using BMI		
		Yes N (%)	No N (%)	Total N (%)
Obese using body fat percentage	Yes	178 (64)	34 (12.2)	212 (76.2)
	No	35 (12.6)	31 (11.2)	66 (23.8)
	Total	213 (76.6)	65 (23.4)	278 (100)

Table 4 depicts the comparison of body fat percentage and waist-hip ratio with BMI in obesity estimation and area under the ROC curve (AUC). In the comparison of body fat percentage, waist-hip ratio with standard BMI found that body fat percentage was fairer than waist-hip ratio in the obesity assessment of diabetic subjects. The AUC of body fat percentage was 0.656 and the AUC of waist-hip ratio was 0.501 and was found to be statistically significant.

**Table 4:** Area under the curve

Test Result Variable(s)	Area	Std. Errors	Asymptotic Sig.	Asymptotic 95% Confidence Interval	
				Lower Bound	Upper Bound
VAR00002 (Body Fat Percentage)	0.656	0.041	0.000	0.575	0.737
VAR00003 (Waist Hip Ratio)	0.501	0.041	0.981	0.421	0.581

## Discussion

In our study, the prevalence of obesity was found to be 76.6% compared to 58.7% in a study conducted by Vasanthakumar, and Kambar [7], 85.8% in a study by AlShahrani et.al. [13], and 71% in a study by Akholkar and Gandhi [14]. The variation in the prevalence of obesity amongst all these studies could be because of differences in the population surveyed and study settings.

When compared to T2DM individuals older than 60 years, obesity was considerably higher in those under 50 years old [15]. In our study also, only 9% of diabetic subjects of age > 60 years were obese as compared to those less than 60 years of age. In a study by Al-Mahroos et al, it was revealed that 20.7% of male and 39.3% of female T2DM patients were obese [16]. In our study also, obesity was more among females (85%) as compared to males (63%). Obesity was more prevalent in the diabetic subjects who had a family history of diabetes (96%) than those subjects who did not have a family history of diabetes (58%) and this was found to be statistically significant. A family history of diabetes was linked to both obesity and T2DM, according to Cederberg et al. [17]. Variable genes control a person's susceptibility to recognized risk factors, which may explain why most diabetics are obese but only a few obese persons suffer from T2DM. Many variant genes that are associated to diabesity have been discovered through genome-wide association studies (GWAS) [18,19].

Out of 39 diabetic bidi/ cigarette smokers, 64% were obese and out of 239 diabetic non-smokers, 78.7% were obese. Thus direct association was not observed in the current study. In contrast, a study by Sulander et al. found that the relative risk of obesity was higher in present light smokers and lower in ex-heavy smokers when compared to non-smokers. Older smokers who smoke heavily are at risk for obesity and diabetes [20]. All hypertensive subjects were obese. Obesity was associated with hypertension and this was found to be statistically significant. Obesity measurements were positively related to diabetes and hypertension at all locations; however, prevalence did statistically vary by site. These correlations were consistent across four regions: Africa, East Asia, South America, and South Asia. [21]. In our study also, obesity was more prevalent in those diabetic participants who were taking high energy-dense food (63%) than those who were not (37%), and this was found to be statistically significant.

In our study, it was found that the total number of obese patients was 213 and 212 out of 278 diabetic subjects as confirmed by applying BMI and body fat percentage formulae, respectively. Similar conclusions were drawn by Shukohifar et al. in their study, where 76.6% of subjects were overweight diabetic participants and 23.4% were normal diabetics. Additionally, 25.7% and 74.3% of the population under study had body fat percentages that were classified as non-fat and fat. According to this study's findings, body fat percentage can be seen as a stronger predictor of hyperglycemia in the male group than BMI, while in the female group, it was a greater predictor of hypertension, hypertriglyceridemia, and low high-density lipoprotein than BMI [22]. In contrast to overall obesity, Ohnishi et al. discovered that central obesity is strongly linked to T2DM risk [23].

The limitation of the study is that due to the cross-sectional study design, the direct association between risk factors and diabesity could not be confirmed. A further case-control study may prove the association between diabesity and risk factors. Step 3, biochemical measurement, of the WHO step-wise approach to chronic disease risk factor surveillance, was not followed.

## Conclusions

Body fat percentage can identify obesity among diabetic individuals who are non-obese by BMI. It is a simple estimation that uses waist circumference in cm and age in years. The present study will be beneficial to non-obese diabetic patients. Behaviour change communication can be administered, thereby reducing insulin resistance and improving compliance and adherence to the treatment. Due to cross-sectional study design, the direct association of risk factors and diabesity cannot be ascertained, further analytical studies need to be conducted to prove it.
